# Human and Machine Learning in Non-Markovian Decision Making

**DOI:** 10.1371/journal.pone.0123105

**Published:** 2015-04-21

**Authors:** Aaron Michael Clarke, Johannes Friedrich, Elisa M. Tartaglia, Silvia Marchesotti, Walter Senn, Michael H. Herzog

**Affiliations:** 1 Brain Mind Institute, École Polytechnique Fédérale de Lausanne (EPFL), Lausanne, Switzerland; 2 Department of Physiology, University of Berne, Berne, Switzerland; 3 Department of Statistics, Columbia University, New York, NY, USA; 4 Centre National de la Recherche Scientifique, Paris, France, Université Paris Descartes, Centre de Neurophysique; 5 Physiologie et Pathologie, Paris, France; 6 Departments of Statistics and Neurobiology, University of Chicago, Chicago, Illinois, United States of America; University of Sheffield, UNITED KINGDOM

## Abstract

Humans can learn under a wide variety of feedback conditions. Reinforcement learning (RL), where a series of rewarded decisions must be made, is a particularly important type of learning. Computational and behavioral studies of RL have focused mainly on Markovian decision processes, where the next state depends on only the current state and action. Little is known about non-Markovian decision making, where the next state depends on more than the current state and action. Learning is non-Markovian, for example, when there is no unique mapping between actions and feedback. We have produced a model based on spiking neurons that can handle these non-Markovian conditions by performing policy gradient descent [1]. Here, we examine the model’s performance and compare it with human learning and a Bayes optimal reference, which provides an upper-bound on performance. We find that in all cases, our population of spiking neurons model well-describes human performance.

## Introduction

Typical laboratory experiments on human learning provide trial by trial feedback following each stimulus presentation. In everyday learning scenarios, however, feedback is often delayed and sparse [2]. In backgammon, for example, several moves must be made before a player receives feedback about a game’s outcome (win, or lose). From this feedback it is impossible to infer directly whether a particular move was good or bad. In addition, rewards might vary from one learning situation to the next. For example, different apples within an orchard might have different tastes. Learning in these situations is well-described by what are known as reinforcement learning (RL) models.

Temporal Difference (TD) methods for RL usually assume that an agent is in one of many discrete states, as in, for example, backgammon [3, 4, 5]. In each state, the agent chooses an action that brings it to a new state until a goal state is reached. Reaching the goal state comes with a reward. When a reward is encountered, the model increases the probability of taking the action immediately leading to the reward and, to a lesser degree, temporally distant actions. The discounting of temporally distant actions can be implemented in terms of an “eligibility trace” [5]. The model can also be modified to handle continuous states and actions, by either discretizing the spaces, or by defining value functions over the spaces that indicate the expected reward for any given state or action [6].

Most RL models assume that the underlying decision process is Markovian, i.e., the next state depends on only the action taken at the current state—history does not play a role (formally, for states *s* and actions *a* at times [*t* + 1, *t*, …, 0]: *p*(*s*_*t*+1_∣*a*_*t*_, *s*_*t*_, *a*_*t*−1_, *s*_*t*−1_, …, *a*_0_, *s*_0_) = *p*(*s*_*t*+1_∣*a*_*t*_, *s*_*t*_)). Many everyday learning scenarios, however, are non-Markovian. An example of a non-Markovian learning situation that prior models have been unable to handle occurs when feedback signals are randomly and independently intermixed, such that the feedback for state *s*_*t*+1_ may occasionally come before that for state *s*_*t*_. This kind of learning situation occurs, for example, when a person learns which course of a three course meal made them sick. This situation is non-Markovian by virtue of the feedback schedule because the learning agent cannot directly measure the reward associated with state *s*_*t*+1_; instead, the reward at state *s*_*t*+1_ can only be estimated by averaging the rewards from states *s*_*t*+1_, *s*_*t*_, *s*_*t*−1_, … over the multiple times at which state *s*_*t*+1_ is encountered.

Non-Markovian situations also occur when an agent is rewarded for traveling from *A* to *B* via *C*, but not when skipping *C* (where *C* is a switch-state). For example, when going on a boat tour, one cannot board the boat without first paying at the ticket counter. This process is non-Markovian because the new state that the agent arrives at, depends on more than the immediately preceding state (i.e., it is non-Markovian by virtue of the state space’s structure). Many RL models do not work well under non-Markovian conditions (see, however, [7]).

We have recently developed a policy gradient method for a population of spiking neurons that is able to learn under several different types of non-Markovian RL situations [8, 9, 1]. The model architecture consists of a three-layer neural network, with an input layer (*X*) a hidden layer (*Y*) and a decision making layer. A unique feature of the model is that the input patterns (*X* activities) and hidden layer (*Y*) activities are represented with populations of spiking neurons. The spikes (delta functions) are input to decaying exponentials (eligibility traces) that cause the network to hold decisions in memory until reward reception. Three such eligibility traces are used, each with different decay rates. The different decay rates allow information to be integrated over the time course of individual spikes, the stimulus duration, and the reward delay respectively. Synaptic weight changes between the *X* and *Y* neurons depend only on the third eligibility trace and the reward at time *t* (see S1 Text).

Not only does this model handle a wide array of Markovian and non-Markovian learning scenarios, but it also proceeds on very few assumptions about the given task. In particular, it does not assume that the task is broken up into discrete episodes, it does not assume that the same actions will always lead to the same states (i.e., it can handle probabilistic state transitions), it does not assume that the reward is received only at an episode’s end, nor does it know that the current experiment’s rewards are single-valued or binary. The model, in fact, goes beyond dealing merely with binary win/loose situations, and can handle any bounded, continuous or discrete, reward distribution.

One crucial question is how such a powerful model compares with human non-Markovian learning. Here, we tested two important non-Markovian tasks: learning with switch-states and learning with intermixed feedback. We compare our model with a simple policy gradient algorithm from the literature [10], and an optimal Bayesian learner. Optimal learning performance is dictated by the task, so we designed custom Bayesian learners for each experiment.

## Results

### Experiment 1: Learning with Switch-States

The first non-Markovian learning situation we consider is learning with switch-states. Here, a goal may be reached only when a key switch-state has been visited first. Traditional reinforcement learning algorithms have difficulty with such tasks due to the Markov assumption that the optimal actions at a given state are independent of the previously visited states.

#### Psychophysics

**Fig 1:**
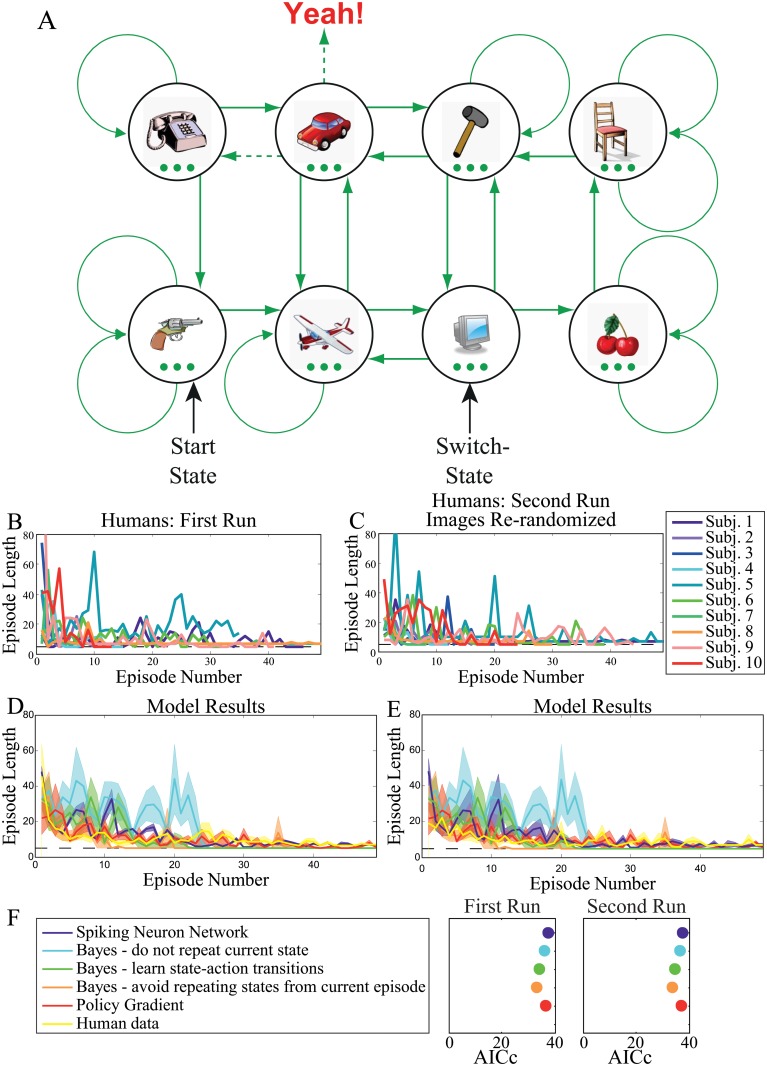
The switch-state experiment. **A**. One image per trial was presented with three green disks below. Participants clicked on one of the disks to proceed to the next image. The first image in an episode was always the same (the pistol in this example). Green arrows indicate the outcomes of the three possible actions for each image. Participants did not know, before training, which disk press led to which new image. The target “Yeah!” could be reached only from one state (the car in this example) and only if the image marked “Switch-State” had been visited first (the monitor in this example). If the switch-state had not been visited first, then, the same action, that brought the participant to the target, would instead bring him or her to a different state (e.g. the telephone in this example). Performance was evaluated by measuring the number of states visited between the start state and goal state as a function of episode number. **B**. First run through the experiment. Human data plotting the number of trials required to complete an episode (episode length) versus episode number. Black dashed lines mark the minimum number of trials (i.e. five) required to successfully complete an episode. Note that we did not average participant data because participants completed differing numbers of episodes. **C**. Second run through the experiment, where the environment structure was the same as shown in A, but different images were randomly assigned to the nodes. Participants learned each task in less than 50 episodes. **D**. Model results for the first run through the experiment. **E**. Model results for the second run through the experiment. All models tested show similar results compared to the human data. **F**. Legend for panels D, E and F Akaike information criterion (corrected for finite sample sizes) for each model. Models with higher ratios between model performance and their number of parameters yield lower AICc values. The Bayesian model that avoids repeating states from the current episode is the best model, although all models performed similarly.

The procedures for experiment 1 are explained in Fig 1 and in the methods (section 6). Participants were presented with an image having three disks below it. A new image was presented when the participants clicked on one of the disks. In the example shown in Fig 1A, the participant started at the “Start State” image. The goal was to reach the “Yeah!”, but in order to do so, the participant first had to visit the state marked “Switch-State”. The sequence of images and actions leading up to and including the goal state constituted one *episode*. Participants completed as many episodes as they could in two sessions of 10 minutes each. In the second session, the images assigned to each node were re-randomized relative to the first session, but they were placed in the same node connection structure as in the first session.

Observers improved their performance from the first to the last episode (Fig 1B). When the images assigned to each node were re-randomized in the second session, participants showed a similar learning pattern to the original experiment, with slightly faster initial learning (Fig 1C).

#### Modeling

The above experiment proves that humans are capable of learning in non-Markovian environments involving switch-states. We next compared human performance with the performance of a spiking neuron model, a Bayes-optimal model that provides an upper limit on learning performance for this particular task, and a policy gradient model.

##### Population of spiking neurons

In the spiking neuron model, decisions are made based on whether the majority of neurons in a population did or did not elicit a spike (see Supplementary S1 Text: Population of Spiking Neurons). Fig 1D plots model performance (purple trace). The model nicely approximates human performance even though it makes very few assumptions.

##### Bayesian learner

In order to quantify human and the spiking network model’s performance, we compared both to an optimal Bayesian model. A detailed explanation of the model can be found in Supplementary S1 Text: Bayesian learning in the switch-state task.

A Bayesian learner uses all available information to make optimal decisions in a given learning task. It is unclear, however, how much information our participants gleaned from the task instructions and how much they brought to the task from their own personal experience. In order to handle this variability, we developed three Bayesian models—the first incorporating a minimal amount of task-relevant knowledge, and the subsequent models encompassing the previous models, while incorporating successively more information.

*Model 1.* Learns directly using the maximum a posteriori (MAP) estimate over all state-action sequences (Fig 1D—cyan trace) and considers the full history of actions when evaluating rewards.

*Model 2.* Takes past states into account and assumes the Markov property for state-action transition dynamics (Fig 1D and 1E—green trace).

*Model 3.* Avoids repeating states that have already been visited in the current episode (Fig 1D and 1E—orange trace).

All of these models provide comparable approximations to the human data. The third Bayesian model provides the smallest residuals, however, this model is a little better than humans at learning the task (Fig 1).

##### Policy Gradient

In order to compare our results with those obtained using a simple policy gradient algorithm, we adopted the model of [1]. This model adjusts its decision policy based on the gradient of the average reward collected over a given amount of time. Its only free parameter is its learning rate, which we optimized to match the performance of human subjects. Results are plotted in Fig 1D and 1E red trace. This algorithm’s outputs show considerable overlap with our second Bayesian model, which learns state-action transition. Details concerning this algorithm’s implementation are provided in Supplementary S1 Text: Policy Gradient.

##### Model Comparison

The spiking neuron network model, the third Bayesian model, and the policy gradient model (Fig 1D and 1E, purple, orange, and red traces respectively) all qualitatively reproduce the human learning data. In order to quantitatively identify the better model, we compared all three models using the L2-norm of the residuals between the human and model data (Table 1, RSS 1 and RSS 2 give the residuals for the first and second runs of the experiment respectively). We used these to compute the Akaike information criterion [11] corrected for finite sample sizes (Table 1, AICc 1 and AICc 2). The AICc indicates which model provides the best fit/parameter number trade-off. Lower values indicate a more parsimonious model (Supplementary S1 Text: Akaike Information Criterion). Here we found the Bayesian model, that avoids repeating states from the current episode, to provide the most parsimonious description of the data (Fig 1F and Table 1).

**Table 1:** Model Fit Summaries.

Model	k	RSS 1	RSS 2	AICc 1	AICc 2
Spiking Neuron Network	1	4319.86	4666.63	37.14	37.53
Bayes—do not repeat current state	0	6449.06	7052.62	35.81	36.26
Bayes—learn state-action transitions	0	4606.83	4943.63	34.13	34.48
Bayes—avoid repeating states from current episode	0	3853.32	4211.16	33.24	33.68
Policy Gradient	1	3828.59	4169.85	36.54	36.96

Fitting results for each model in the switch-states experiment showing the number of free parameters (k), the residual sums of squares (RSS), and the Akaike Information Criterion adjusted for finite sample sizes (AICc) for runs one and two of the experiment.

### Experiment 2: Intermixed Feedback

#### Psychophysics

**Fig 2:**
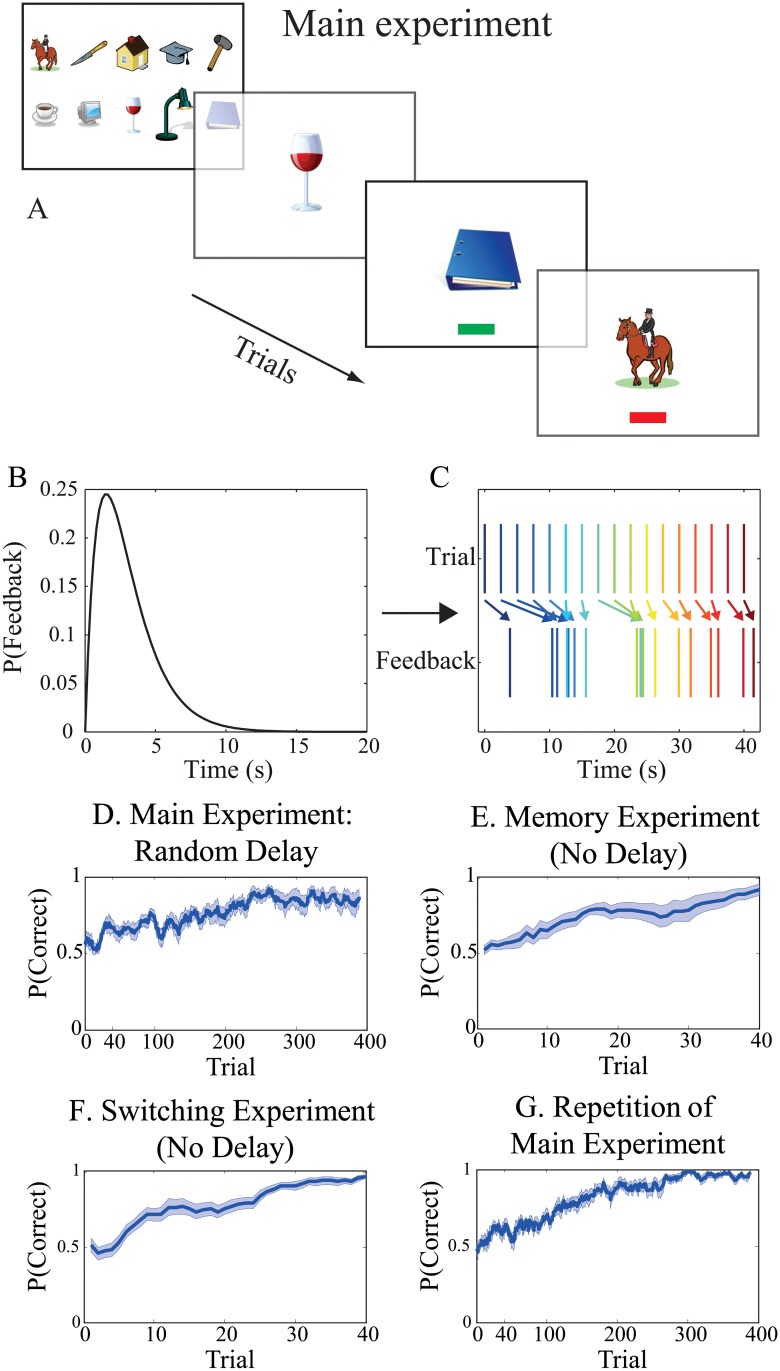
Intermixed feedback experiment. **A**. Participants were first shown the ten images in the learning set. Following this, one image at a time was presented. Each image belonged to either the “left” or “right” category and participants were asked to learn to which category each image belonged. Incorrect and correct responses were followed by feedback in the form of a red or green bar, respectively, at the bottom of the screen. Feedback was randomly delayed and intermixed using the *γ*-probability density function shown in **B**. **C**. Under this probability density function, feedback for a given image could appear after that for the next image. **D**-**G**. Proportion correct (averaged over a 10 trial sliding window) plotted versus trial number (n = 14). **D**. Random delay. Average participant performance improved over 400 trials. **E**. Memory experiment. With immediate feedback, subjects learn within 40 trials, demonstrating sufficient memory capacity for the task. **F**. Switching experiment. This experiment demonstrates that participants can flexibly learn new associations in a short time interval. **G**. In the repetition of the main experiment with 10 new images, learning was more efficient than in the first run of the main experiment (D).

Switch-states are one example of a non-Markovian situation. Another non-Markovian situation occurs when the feedback for different actions are not unique. Here we tested participants’ ability to learn under non-unique feedback. The procedures for this experiment are illustrated in Fig 2A. Participants were first presented with the full set of experimental images. They were instructed that each image belonged to either category one (left button press) or category two (right button press). Next, participants were presented with one image at a time and pressed either the left or the right button. Before learning, participants were unaware of which buttons went with which images. Learning in this paradigm is difficult, therefore, we split up the experiment into two practice tasks with gradually increasing difficulty levels. We first had participants learn to make left/right button presses for four images with immediate feedback, and then we had participants do the same task with randomly intermixed feedback. After this initial training period, we used 10 images with randomly intermixed feedback (Fig 2A). We next examined performance in three additional conditions. First, to establish an upper bound on learning, we used the same procedures as in our main experiment, but with immediate feedback. Second, to determine whether participants can switch the response classifications they previously learned, we replicated the main experiment, but half of the images switched their left/right response classification. Third, to ensure the robustness of our results we repeated the main experiment, but with 10 new images. Further procedural details can be found in the Methods section.

For the main experiment, with 10 images and randomly delayed feedback, participants’ performance improved over the course of 400 trials. This shows that participants are able to learn under a difficult type of non-Markovian learning situation.

For the repetition of the main experiment with 10 new images, and immediate feedback (Fig 2F), all participants reached a very high proportion correct within only 40 trials. This result provides an upper-bound on learning performance for this task. Furthermore, it shows that learning 10 image/action pairings is not beyond the participants’ abilities, and that our subjects had a sufficient memory capacity to learn the task quickly.

For the repetition of the previous experiment with immediate feedback, where for half of the images, we switched the response category (left to right and vice versa), all participants reached 100% correct performance by the end of the 40 trial training period. This result shows that all participants were able to switch previously learned image/action pairings.

For the final repetition of the main experiment with delayed feedback but with 10 new images, we tested the effects of task-familiarity. At this point all participants had familiarized themselves with the main task and they were able to improve performance even more quickly than in the original main experiment (Fig 2E). This shows that task-familiarity can enhance learning performance.

Taken together, our results show that humans are capable of learning under non-Markovian conditions of randomly intermixed feedback.

#### Modeling

##### Population of spiking neurons

**Fig 3:**
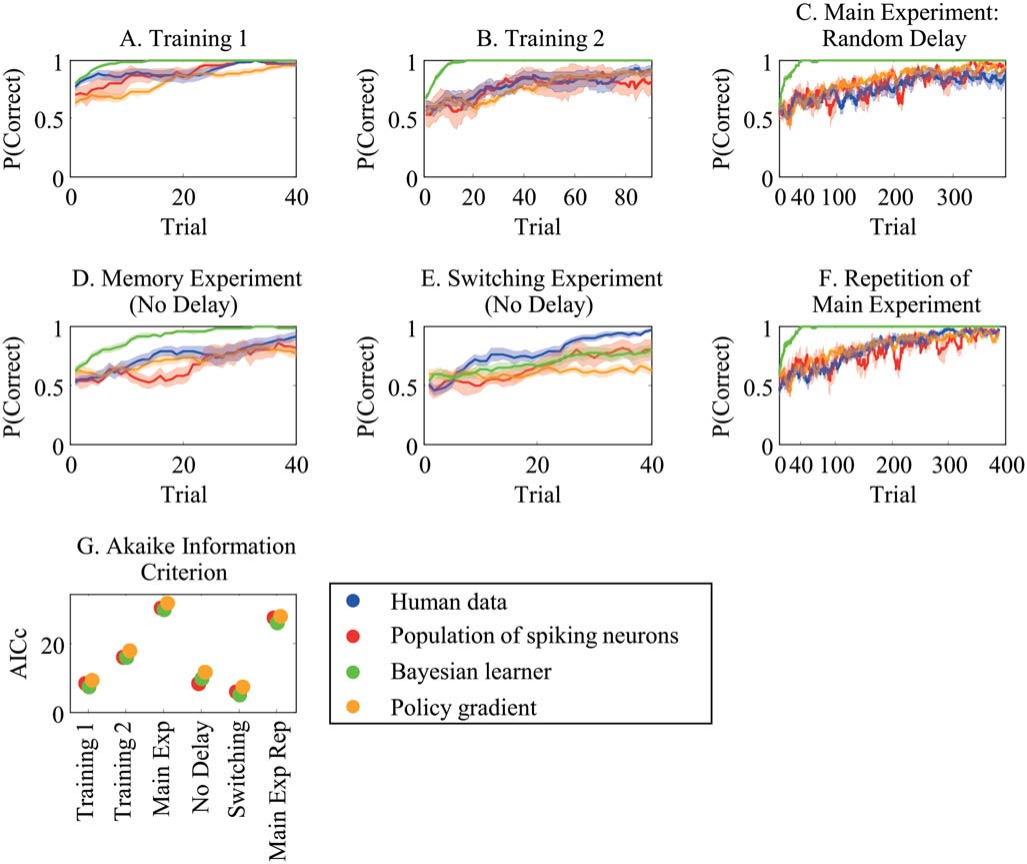
Intermixed feedback experiment human data with model predictions. Proportion correct versus trial number plotted for simulation results from a population of spiking neurons (red), a Bayesian learner (green), and a policy gradient learner (orange) compared with human performance (blue; re-plotted from Fig 2). **A** First training experiment without any delays. **B** Second training experiment with delay. **C**. Main experiment with random delay. **D**. Memory experiment with no delay. **E**. Switching experiment with no delay. **F**. Repetition of the main experiment. **G**. Akaike information criterion (corrected for finite sample sizes) for each model under consideration in the intermixed reward task. Lower AICc values imply greater support for the given model. In general, all models perform similarly.

We ran simulations using the population of spiking neurons model (Supplementary S1 Text: Population of Spiking Neurons). Fig 3A, 3B, 3C, 3D, 3E, and 3F plot mean performance (±SEM) over four runs for the intermixed feedback experiment. The model results are comparable to human results in all variations of the experiment (although possibly less so in Fig 3E).

##### Naïve Bayesian learner

This paradigm differs substantially from that used in experiment one and, subsequently, requires a new Bayesian learner model (S1 Text). In brief, the model puts a prior (Dirichlet Process) over the delay distribution and updates the posterior, whenever new data is observed. For decision making it marginalizes over all delay distributions. Since the feedback is intermixed relative to the responses, the model estimates the delay distribution that best explains the received rewards.

This model provides comparable results to the human data in all cases (Fig 3), suggesting that humans perform roughly optimally in this paradigm.

##### Policy Gradient

Here, we again compare human performance with a simple policy gradient algorithm, using the model of [1]. As for the previous learning scenario, we optimized the algorithm’s learning rate in order to best match the human data. This algorithm thus has one free parameter.

##### Model Comparison

We again computed the L-2 norm of the residuals between the human data and model predictions and used these to compute the AICc for each model (Fig 3G and Table 2). The results indicate that the all models do a similarly good job at predicting human performance.

**Table 2:** Model Fit Summaries.

	Spiking Neuron Network	Bayesian Learner	Policy Gradient
k	1	0	1
Training 1:			
RSS	27.13	28.94	28.98
AICc	8.32	7.39	9.64
Training 2:			
RSS	40.37	44.79	44.13
AICc	16.27	16.13	18.05
Main Exp:			
RSS	84.19	92.39	90.44
AICc	30.97	30.61	32.40
No Delay:			
RSS	27.35	32.63	32.39
AICc	8.48	9.79	11.87
Switching:			
RSS	24.06	25.95	25.90
AICc	5.92	5.21	7.39
Main Exp Rep:			
RSS	72.00	74.67	74.59
AICc	27.84	26.35	28.55

Fitting results for each model in the intermixed feedback experiment showing the number of free parameters (k), the residual sums of squares (RSS), and the Akaike Information Criterion adjusted for finite sample sizes (AICc).

## Discussion

Animals and humans can learn from sparse and intermixed feedback. Reinforcement learning models effectively explain learning in Markovian situations, that is, situations where the outcome of an action depends only on the current state. For example, navigation [12, 13, 14, 15, 16, 17, 18, 19], sequence learning [20, 21], or gambling [22, 23, 24, 25, 26, 27, 28, 29, 30, 31, 32, 33, 34, 35]. But often learning situations are non-Markovian, as when playing chess and the game’s outcome is revealed only after several moves have been made. Recently, however, we showed that classical reinforcement learning models can be extended to cope with non-Markovian learning situations [8, 9, 1]. Here we examined the quality of this model’s description of human learning performance.

To this end, we devised two classes of non-Markovian experiments which are prototypical. One class used switch-states, where a goal state could only be reached when the switch-state had previously been visited. The other class used intermixed feedback, where the reward associated with a particular action could only be estimated by averaging reward signals from multiple past actions. Humans can learn under both types of non-Markovian situations. Performance was well-described by the population of spiking neurons model in both experiments, roughly matching the fits of the other algorithms tested.

Several papers have attempted to model non-Markov decision processes in machines, but [2] and our current paper represent the first to model them in human learning.

Finally, our population of spiking neurons model represents a biologically plausible model with very limited assumptions that performs our reinforcement learning tasks on par with Bayesian and policy gradient learning algorithms. It is not only capable of learning under the non-Markovian scenarios presented here, but it can also learn probabilistic state transition, probabilistic rewards of different sizes at various times and even non-episodic tasks [8, 9, 1]. None of the Bayesian models we examined here demonstrated this level of flexibility and they all made more assumptions about the task structure (although this was done intentionally in order to take into account that subjects might heed task instructions to varying degrees). We thus assert that our biologically plausible population of spiking neurons model is more akin to the learning machinery of the human brain: it is extremely versatile, capable of learning many different classes of tasks, while making minimal assumptions about the tasks’ structure and closely predicting human performance on par with less biologically plausible models.

## Materials and Methods

### Participants

A total of 21 students from the École Polytechnique Fédérale de Lausanne (EPFL) participated. Written informed consent was obtained from each participant prior to the experiment. Participants were paid 20 Swiss Francs/hour after completing the experiment. Prior to the experiment, the participants were informed about the study’s general purpose and were told that they could quit at any time. All procedures conformed to the declaration of Helsinki and were approved by the Centre hospitalier universitaire vaudois ethics committee (Protocol 259/07: Basic aspects of object recognition).

### Setup

Experiments were conducted on a 2.8 GHz Intel Pentium 4 processor workstation running Windows XP. A Phillips 201B4 monitor, running at a screen resolution of 1024 × 768 pixels and a refresh rate of 100 Hz was used for stimulus display. Experiments were scripted in Matlab 7.11 using custom software and extensions from the PsychToolbox for Windows XP [37, 38].

### Procedures

#### Switch-States Experiment

Ten people participated in this experiment. The experiment began with a screen showing eight images. These images were used throughout the experiment. Participants were instructed that throughout the experiment, they would be presented with one image at a time and that in order to proceed to the next image they had to make a mouse click on one of three green disks presented below the image (Fig 1A). At the trial sequence’s end a “Yeah!” appeared as the final image. The participants were instructed that their goal was to reach the “Yeah!” as often as possible within 10 minutes. They were further informed that the associations between disks and images would not change throughout the experiment. It was initially unbeknownst to the participants that, in order to reach the goal image, they had to pass through the image marked “Switch-State” in order to have access to the goal, as shown in Fig 1A. This constituted their learning task. If participants tried to go directly to the goal without visiting the switch-state image then they were re-directed to another image (the telephone in Fig 1). The state-action space contained both recurrent and outward-bound connections.

There were two runs of 10 minutes each, where participants completed as many episodes as they could in the allotted time. The number of images visited per episode was recorded as a function of episode number. From the first 10 minute run to the second, the images assigned to each node were re-randomized, but the underlying node-connection structure remained the same. The effects of a left/middle/right disk press were randomized over images such that a left disk press might lead to a downward traversal in the graph for one image, but a rightward traversal for another image.

#### Intermixed Feedback Experiment

Fourteen participants (including three from the previous experiment) learned image-classification pairings (with two possible classifications, left or right, per image) with *randomly intermixed* feedback. This task is inherently difficult, so we built up task-difficulty gradually. The general procedures for these experiments are illustrated in Fig 2.

*Part I: Training with immediate feedback*

We started with a basic task that involved learning of only four images (Fig 2A). Participants received immediate feedback about the correctness of their classifications after each image presentation. Feedback was provided in the form of a red or green bar presented at the bottom of the screen indicating an incorrect or a correct response respectively. This step acquainted the participants with the basic task.

*Part II: Training with intermixed feedback*

Next, we replaced the original four images with four new images. We increased task difficulty by randomly delaying the feedback time (*t*) for each image according to a *γ*-probability density function (Eq 1 and Fig 2B) with a shape parameter of *k* = 2 (which controls the function’s width) and a scale parameter of *θ* = 1.5 seconds (which determines the function’s peak location):

$$\begin{array}{c} p\left(t|\theta ,k\right)=t^{k-1}\frac{exp(-t/\theta )}{\Gamma \left(k\right)\theta^{k}}. \end{array}$$ (1)

Where Γ is the gamma function: $\Gamma (z)=\int_{0}^{\infty }e^{-t}t^{z-1}\text{d}t$. This scenario allowed feedback for the participants’ to go out of order with respect to the image order (Fig 2B and 2C). The task was to learn the correct classifications (left or right) that the computer had randomly assigned to each image. Here, it was possible that the feedback for an image could have been delayed to the point where it was presented simultaneously with a later image.

*Part III: Main experiment*

We increased task-difficulty by requiring participants to learn image-classification pairings for 10 images (instead of 4), again with two classifications per image and with randomly intermixed feedback following a *γ*-probability density function with the same parameters as in *part II* (Fig 2B).

*Part IV: Memory control-experiment*

To test the limits of our participants’ basic associative memory capacity we had them learn image-classification pairings for 10 new images with immediate feedback.

*Part V: Switching control-experiment*

We examined participants’ efficacy at reversal learning, that is, learning where some of the images switched which of their classifications were rewarded. Here, participants repeated the Memory experiment, but with half of the images having swapped classification categories (right to left and vice versa).

*Part VI: Replication of the main experiment*

Here, we replicated the main experiment of *Part III* with 10 new images.

### Analysis

The proportion of correct classifications across trials was calculated by convolving the sequence of correct and incorrect responses with a convolution kernel that averaged responses over 10 trials at a time. This trace was then averaged over participants to generate Figs 2D–2G and 3A–3F.

## Supporting Information

S1 Text Details of model implementation. Here we provide details of our population of spiking neurons model, our Bayesian learners, and our policy gradient algorithm, and the Akaike information criterion that we used to evaluate them.
(PDF)

S1 Fig Example of a generated tree. Here in the first episode the learner already tried making a left button press (*l*), which was terminal and yielded no reward, hence the probabilities have been updated to *P*(*C*∣(*l*)) = 0 and $P(R|\bar{C},(l))=0$. In the second episode the sequence (*r*, *l*, *l*) (right-left-left) has been performed yielding no reward either. Because (*r*,*l*,*l*) includes the non terminal sub-sequences (*r*) and (*r*,*l*), after the second episode the posteriors *P*(*C*∣*s*) and $P(R|\bar{C},s)$ for *s* ∈ {(*r*), (*r*,*l*), (*r*,*l*,*l*)} are updated: *P*(*C*∣(*r*)) = *P*(*C*∣(*r*,*l*)) = 1, *P*(*C*∣(*r*,*l*,*l*)) = 0 and $P(R|\bar{C},(r,l,l))=0$. All other probabilities still have their prior values. Now the learner has to decide on the best sequence to perform in the third episode, which is done through Monte Carlo tree generation. Starting at the root, it draws from each of the distributions *P*(*C*∣(*l*)), $P(R|\bar{C},(l))$ and *P*(*C*∣(*r*)) a sample with the result that the sequence (*l*) terminates without reward while the sequence (*r*) continues. Thus no rewarded sequence of length one has been sampled and the growing of the tree continues. Because sequence (*l*) was terminal, only further sequences starting with *r*, namely (*r*,*l*) and (*r*,*r*), need to be considered. The random drawing of outcomes yields that (*r*,*l*) continues while e.g. sequence (*r*,*r*) terminates with reward *R*. Hence the counter of sequence (*r*,*r*) is incremented by one. A rewarded path of length two has been found and the tree is not grown any deeper. Given the current values of the probabilities *P*(*C*∣*s*) and $P(R|\bar{C},s)$ thousands of such trees are generated. In the end, the sequence with the highest counter is the MAP estimate of the best sequence.
(EPS)
