# Supplementary material for: Human and Machine Learning in Non-Markovian Decision Making
Source: PLoS One. 2015 Apr 21;10(4):e0123105. doi: 10.1371/journal.pone.0123105 (PMC4405578; doi:10.1371/journal.pone.0123105)
Supplement: S1 Text — Here we provide details of our population of spiking neurons model, our Bayesian learners, and our policy gradient algorithm, and the Akaike information criterion that we used to evaluate them. (PDF) [file pone.0123105.s001.pdf]

Supporting Information

Raw data for this manuscript can be accessed at:
`https://osf.io/9sacv/?view_only=187a993a964342cfab1e8f7f65678fa9`.

1.1 Population of Spiking Neurons

For our population of spiking neurons model we used a network of Spike-Response-Model (SRM) neurons with escape noise and a feed-forward architecture [39]. The input to the model is a set of spike trains encoding the environment's current state (i.e., the currently presented image in the intermixed reward task, or the currently presented and the previously presented images in the switch-state task). The output neurons are made up of sub-populations, where each sub-population receives inputs from a randomly selected sub-set of input neurons, and where each sub-population codes for a different action. Hence, the number of sub-populations equals the number of actions. At the end of each stimulus presentation each sub-population's output activities are read-out by decision making circuitry that makes behavioral decisions based on the information monitored from the sub-population activities during the stimulus presentation period. Rewards may be received immediately following an action performance, or with some variable delay. Decisions are made in a probabilistic winner-take-all fashion, where the probability of taking an action associated with a given sub-population increases with the number of spikes fired by the neurons in that sub-population. The synaptic weights between the input neurons and the output sub-populations are then updated according to a multi-factor plasticity rule, which optimizes the received rewards by performing a stochastic gradient ascent on the expected reward rate. Plasticity induction is based on a cascade of synaptic memory traces. The traces correlate presynaptic input first with postsynaptic events, next with the behavioral decisions and finally with the external reinforcement:

$$\tau_M \dot{E}_1 = -E_1 + \sum_{s \in x_t} \frac{1}{\tau_S} e^{-(t-s)/\tau_S} \quad (2)$$

$$\tau_D \dot{E}_2 = -E_2 + E_1(t)\,\mathrm{post}_1(t) \quad (3)$$

$$\tau_R \dot{E}_3 = -E_3 + E_2(t)\,\mathrm{post}_2(t)\,\mathrm{Dec}(t) \quad (4)$$

$$\dot{w} = E_3(t)\,\mathrm{Rew}(t)\,, \quad (5)$$

The first synaptic eligibility trace E_1 bridges the gap between the synaptic time scale τ_S and the membrane time constant τ_M by low pass filtering. The second eligibility trace E_2 is a synaptic buffer roughly encoding the covariance between the past pre- and post-synaptic activity relevant for learning.

The third eligibility trace E_3 captures the interactions between single neuron activity, the population activity and the behavioral decision. The final stage of the cascade remodulates E_3 by the reward signal to yield the synaptic weight update.

Model details

Here we used the model of [1] with a few modifications. For example, we required three output populations instead of two, in order to accommodate the ternary decisions of the switch-state task. We also used a larger network with 100 neurons and an input dimension size of 250 neurons (this is the model's only free parameter). The increased input dimension size relative to [1] was compensated for by reducing the input Poissonian spike rate from 6 to 2 Hz. The current image state was encoded by 150 input spike trains and an additional 100 spike trains coded for the previous image state.

Focusing on one neuron, the input $\mathbf{X}$ is a spike pattern made up of M spike trains that yield an

output spike train Y. The membrane potential u can be written as:

$$u(t) = u_0 + \sum_{i=1}^{M} w_i \sum_{s \in X_i} \epsilon(t-s) - \sum_{s \in Y} \kappa(t-s)\,. \tag{6}$$

The postsynaptic kernel $\epsilon(t)$ and the reset kernel $\kappa(t)$ vanish for $t \leq 0$. For $t > 0$ they are defined as:

$$\epsilon(t) = \frac{1}{\tau_M - \tau_S}\left(e^{-t/\tau_M} - e^{-t/\tau_S}\right) \text{ and } \kappa(t) = \frac{1}{\tau_M} e^{-t/\tau_M}\,.$$

For the resting potential we use $u_0 = -1$ (arbitrary units). Further, the membrane time constant is $\tau_M = 10\,\text{ms}$ and the synaptic time constant is $\tau_S = 1.4\,\text{ms}$. Action potential generation is controlled by an instantaneous firing rate $\phi(u)$ which increases with the membrane potential. At each time point t, the neuron fires with probability $\phi(u(t))\delta t$ where δt represents an infinitesimally small time window (we use $\delta t = 0.2\,\text{ms}$ in our simulations). Our firing rate function is:

$$\phi(u) = k e^{\beta u},$$

where $k = 0.01$ and $\beta = 5$. These values are based on those reported experimentally by [40] where one takes our unit-less resting potential of -1 and threshold of 0 to correspond to the biological values 70mV and 50mV, respectively.

As mentioned, our input layer has $M = 250$ neurons and there are n output populations (each denoted as Pop_i, $i \in \{1, 2, ..., n\}$), of $N = 100$ neurons each, which code for individual decisions D_i. We deliberately fixed the network size to 100 neurons. The only free parameter optimized to fit the data was the input dimension. Using a grid search for the input dimension, we found that a value of 250 minimized the residual sum of squares between data and model predictions. For the chosen input dimension, the learning rate was obtained via a grid search, however, it was not optimized to fit the data, hence it does not add a further free parameter, but it was optimized for general learning performance such that the number of errors accumulated over all trials was minimized. In our formulation, each input layer neuron synapses onto an output sub-population neuron with a probability of 80%, leading to many shared input spike trains between the neurons. The output sub-population responses are read out by the decision making unit based on a spike/no-spike code. We introduce the coding function $c(Y^\nu)$, with $c(Y^\nu) = -1$, if neuron ν does not spike and $c(Y^\nu) = 1$ if it does. The population activity A_i being read out by the decision making unit is:

$$A_i(\mathbf{Y}) = \frac{1}{\sqrt{N}} \sum_{\nu \in \text{Pop}_i} c(Y^\nu).$$

Normalizing the activity by $\sqrt{N}$ ensures that $\text{Var}(A) = \mathcal{O}(1)$, thus being of the same order as the noise in the decision readout process. Using this activity readout, and denoting the vector of all activities as $\mathbf{A} = (A_1, ..., A_n)$, the behavioral decision D is made stochastically, with the likelihood $P(D = D_i|\mathbf{A})$ given by the softmax function:

$$P(D = D_i|\mathbf{A}) = \frac{\exp(A_i)}{\sum_{j=1}^{n} \exp(A_j)}\,. \tag{7}$$

We now describe the terms modulating synaptic plasticity. As shown in [41], the probability density, $P_w(Y)$, that a neuron produces an output spike train Y in response to a stimulus $\mathbf{X}$ during a decision period lasting from $t = 0$ to $t = T$ satisfies:

$$\ln P_w(Y) = \sum_{s \in Y} \ln \phi(u(s)) - \int_0^T \mathrm{d}t\, \phi(u(t)).$$

The first postsynaptic signal is:

$$\mathrm{post}_1(t) = -k\,\beta\, e^{\beta u(t)} + \beta \sum_{s\in Y} \delta(t-s). \tag{8}$$

With this choice, the second eligibility trace E_2 (from Eq. 3) is an approximation to the derivative of $\ln P_w(Y)$ with respect to the strength of synapse i, a quantity known as *characteristic eligibility* in reinforcement learning [42].

The second postsynaptic signal (from Eq. 4) is given by:

$$\mathrm{post}_2(t) = \mathrm{sign}(C(t) - \vartheta)\,,$$

where, C is a concentration variable that reflects neural spiking. C is set to 1 each time there is a postsynaptic spike, and otherwise C decays as $\tau_D \dot{C} = -C$. The magnitude of C is compared to a threshold ϑ, which we set to $\vartheta = e^{-1.1}$ in our simulations.

The decision feedback $\mathrm{Dec}(t)$ used in Eq. (4) is encoded by means of a variable c_{Dec}, representing the concentration of an ambient neurotransmitter, such as dopamine [43, 44, 45]. The feedback manifests as a temporary change in the neurotransmitter's production rate. The value of $\mathrm{Dec}(t)$ is determined by the derivative of $\ln P(D_j|\mathbf{A})$ with respect to A_i [1] and, given Eq. (7), this derivative is simply $\delta_{ij} - P(D_i|\mathbf{A})$. Thus, c_{Dec} evolves with time as:

$$\tau_{\mathrm{Dec}}\dot{c}_{\mathrm{Dec}} = -c_{\mathrm{Dec}} + c^0_{\mathrm{Dec}} + (\delta_{ij} - P(D_i|\mathbf{A}))\,\Theta(t; nT, L_{\mathrm{Dec}}).$$

In this equation, nT denotes the time at which the stimulus ended, thereby evoking the population activity $\mathbf{A}$ and a behavioral decision D_j. Here, the step function $\Theta(t; nT, L_{\mathrm{Dec}})$ equals 1 if $nT \le t \le nT + L_{\mathrm{Dec}}$, and is otherwise zero. Parameter values in the simulations are $\tau_{\mathrm{Dec}} = 10\,\mathrm{ms}$ and $L_{\mathrm{Dec}} = 50\,\mathrm{ms}$. The above equation holds up to time $(n+1)T$ when the subsequent stimulus presentation ends, at which point the decision variables D and $\mathbf{A}$ are replaced by their values for the latter stimulus. The decision feedback $\mathrm{Dec}(t)$ is simply:

$$\mathrm{Dec}(t) = c_{\mathrm{Dec}}(t) - c^0_{\mathrm{Dec}}\,.$$

The reward feedback $\mathrm{Rew}(t)$, modulating synaptic plasticity in Eq. (5), is encoded in the concentration c_{Rew} of a neurotransmitter, e.g. dopamine. Up to the point in time s' when further reinforcement becomes available, the concentration variable evolves as:

$$\tau_{\mathrm{Rew}}\dot{c}_{\mathrm{Rew}} = -c_{\mathrm{Rew}} + c^0_{\mathrm{Rew}} + R_{\mathrm{s}}\,\Theta(t; s, L_{\mathrm{Rew}})\,.$$

Here the step function $\Theta(t; s, L_{\mathrm{Rew}})$ equals 1 if $s \le t \le s + L_{\mathrm{Rew}}$, and is zero otherwise. Parameter values in the simulations are $\tau_{\mathrm{Rew}} = 50\,\mathrm{ms}$ and $L_{\mathrm{Rew}} = 50\,\mathrm{ms}$. The reward feedback read-out is determined by the deviation of the current neurotransmitter level $c_{\mathrm{Rew}}(t)$ from its homeostatic value and equals

$$\mathrm{Rew}(t) = \eta\left(c_{\mathrm{Rew}}(t) - c^0_{\mathrm{Rew}}\right)\,.$$

The parameter η is the positive learning rate which, for notational convenience, we absorb into the reward signal.

1.2 Bayesian learning in the switch-state task

Whereas the population of spiking neurons is oblivious to the task's structural aspects, the human subjects are given explicit instructions about the task structure. We incorporate this prior knowledge into a Bayesian learner, thus, tailoring it to the specific task at hand. The learner tries to maximize its reward rate, i.e., it tries to find a *shortest* rewarded sequence of actions. A sequence s of actions is a list

of actions $(a_1, a_2, ..., a_n)$ where a_1 is the first and a_n the last action. An action a_i is one out of the three available actions in the task, e.g. “clicking on the left disk”. Some of the realizable sequences continue (C), while the others terminate $(\bar{C})$. For example, in Fig. 1A, starting at the “Start State” and taking the action that leads to the next image to the right is an action that continues. If the learning agent is at the car and chooses the action that leads up to the “Yeah!” image, then this is an action that terminates. In the former case no reward is obtained, $P(R|C, s) = 0$, whereas in the latter case the terminal sequence yields reward (R). Due to the instructions given to the human subjects, the learner has the prior knowledge that state transitions and rewards are deterministic (clicking on the same disk for a given image will always lead to the same subsequent image). Before encountering a sequence s during the game the agent has some prior beliefs about whether the sequence will result in a reward. These beliefs are appropriately quantified by probabilities. Initially the probability that the sequence continues and the conditional probability of reward given that the sequence ends are set to some prior probability values, $P(C|s) = p_c$ and $P(R|\bar{C}, s) = p_r$. We want to stress that these probabilities quantify the Bayesian learner’s beliefs and do not describe stochasticity in the state transitions or rewards which were not present in the current experiments. Over episodes the beliefs $P(C|s)$ and $P(R|\bar{C}, s)$ about encountered sequences s are updated according to Bayes rule. Based on the posteriors one can calculate the likelihood that a sequence is a *shortest* rewarded sequence. This calculation involves a Monte Carlo procedure where potential shortest rewarded sequences are sampled and the next sequence is determined by selecting the one that was sampled most often, which corresponds to the maximum a posteriori (MAP) estimate

The Monte Carlo process used to determine the probability that an action sequence is in the set of shortest rewarded sequences proceeds as follows. We construct a decision tree based on $P(C|s)$ and $P(R|\bar{C}, s)$ (see S1 Fig.). A draw from $P(C|s)$ determines whether the branch continues (C) (i.e. s is a node) or whether it ends $(\bar{C})$ (i.e. s is a leaf).[1] Terminal sequences correspond to leaves of the tree. For leaves, a draw from $P(R|\bar{C}, s)$ indicates whether the sequence is rewarded (R) or not $(\bar{R})$. Nodes correspond to unfinished episodes and are never rewarded, $P(R|C, s) = 0$. The tree’s depth is increased until at least one leaf is a rewarded sequence. At this stage the rewarded leaves are the shortest rewarded sequences. Because we are only interested in these, it is not necessary to grow the tree any further. We generate thousands of such trees and count, for each sequence, how often it is a shortest rewarded sequence. The sequence with the maximal count is the maximum a posteriori (MAP) estimate. If two or more sequences have the same count then one sequence is selected randomly and uniformly as the estimate.

If a sequence s is not terminal then the learner picks the highest probability sequence from the remaining sequences that start like the previously selected sequence s and so on until the sequence does not continue any further. For example, if the first selected sequence is $(right)$ and not terminal, then the learner picks from the sequences $(right, ...)$, all of which start with $right$.

In the switch-state experiment, episodes continue until the reward is obtained, hence $p_r = 1$. For the prior on continuing we used $p_c = 0.5$. For sequence selection we considered three different methods, with each subsequent method making more assumptions than the last.

1.3 Bayesian learning of stimulus-response associations with randomly intermixed reinforcement

Here we present the details of our Bayesian learner model employing a Dirichlet Process (DP), first introduced by [46], as a prior over the delay distribution. We assume some familiarity of the reader with DPs (a good introduction is given by [47]).

[1] Note that here we assume that a sequence either always deterministically continues or ends, thus p_c only characterizes the uncertainty prior on encountering the sequence. Pushing the Bayesian paradigm even further, one could introduce a distribution over p_c, the conjugate prior being a beta distribution, update the distribution whenever the sequence is encountered and integrate it out for the decision making.

We make the assumption that the obtained rewards are independent when conditioned on the images' classification labels, which is known as naïve Bayes. Because the learner does not know the parametric form of the delay distribution, we use a flexible non-parametric method [48]. More precisely, we use a Dirichlet Process (DP) [46] as a prior over the delay distribution. Image label assignments ("left", or "right" button presses) are made by calculating the posterior probability distribution for the presented image's label, which involves marginalizing over the other images' labels as well as the delay distribution, and using the maximum a posteriori (MAP) estimate. As a base distribution for the DP we used a Cauchy distribution, motivated by the fact that it is asymptotically scale free. The DP's concentration parameter cancels due to marginalization in the decision making (for a proof see the Supplementary S1.3.5 Text: Posterior probability of the classification vector).

1.3.1 Notation

The data $\mathbf{D}^n$ is a vector of reward and reward-time pairs $\{R_j, t_j\}$ with $j \in \{1, ..., n\}$ where t_j denotes the time at which the jth reward $R_j = \pm 1$ was received. The jth pair is denoted by D_j. Often, the index n is skipped for notational compactness. Time (T) is measured in stimulus durations, where for one stimulus the duration is $T = 1$. The image ending at time t is $x_t \in \{1, ..., m\}$, where m is the number of images. The decision made at time t is $d_t = \pm 1$. The classification labels (left or right button presses) for each image may be collected into a single vector $\mathbf{c} = (c(1), ..., c(m)) \in \{-1, 1\}^m$, which is an m-dimensional binary vector where the component $c(x)$ is the classification label for image x. The probability density distribution for the delay τ is $p(\tau|\boldsymbol{\pi}, \boldsymbol{\mu})$, where $\boldsymbol{\pi}$ and $\boldsymbol{\mu}$ are, possibly infinite-dimensional, vectors parameterizing the distribution.

1.3.2 Decision making

We calculate the posterior probability $P(\mathbf{c}|\mathbf{D})$ of the classification vector $\mathbf{c}$ given the observed data. Classification decision are then made by marginalizing over the other classes $\mathbf{c}^{\backslash y}$:
$P(c(y)|\mathbf{D}) = \sum_{\mathbf{c}^{\backslash y}} P(\mathbf{c}|\mathbf{D})$ (where $\backslash y$ means "not y"), and using the MAP image class estimate: $\arg\max_{c(y)} P(c(y)|\mathbf{D})$ as the response to image y.

1.3.3 Naïve Bayes

We approximate the data as being conditionally independent, $p(\mathbf{D}^n|\mathbf{c}) = \prod_{j=1}^{n} p(D_j|\mathbf{c})$. This strong, and in this task unsatisfied, independence assumption is known as *naïve Bayes* (the independence assumption is unsatisfied because naïve Bayes ignores that there is a bijection between decisions and rewards, i.e. it assumes that one decision could result in multiple rewards). Though in most cases the assumptions are not satisfied, naïve Bayesian algorithms perform amazingly well in practice.

The probability density for observing data point D_j, i.e. reward R_j at time t_j, is:

$$p(D_j|\mathbf{c}, \boldsymbol{\pi}, \boldsymbol{\mu}) = \sum_{s=1}^{\lfloor t_j \rfloor} \delta_{d_s c(x_s), R_j} p(t_j - s|\boldsymbol{\pi}, \boldsymbol{\mu}) \tag{9}$$

- The Kronecker delta ($\delta_{d_s c(x_s), R_j}$) equals one if the reward R_j is provided for the decision (d_s) about the classification ($c(x_s)$) at time s, and is zero otherwise.
- The second term $p(t_j - s|\boldsymbol{\pi}, \boldsymbol{\mu})$ is the probability density specifying the probability that reward R_j for the decision at time s is delivered with delay $t_j - s$.

The symbol $\lfloor \rfloor$ indicates the floor function.

1.3.4 Dirichlet process mixtures

Instead of parameterizing the distribution for the delay τ (e.g. Equation 1) and defining priors over the parameters, one can directly define a prior over the delay distribution, i.e. a measure on measures. A Dirichlet process (DP) is such a distribution over distributions. A draw from it yields a discrete distribution and in order to perform density estimation one convolves the sampled distributions with some kernel K, which gives rise to Dirichlet process mixtures. In a Dirichlet process mixture, we draw the parameters of a mixture model from a draw from a DP:

$$G \sim \mathrm{DP}(G; G_0, \alpha) \tag{10a}$$

$$\mu \sim G(\mu) \tag{10b}$$

$$\tau \sim K(\tau; \mu) \tag{10c}$$

where $\alpha > 0$ is the concentration parameter (such that we observe $\alpha_i - 1$ successes for class i), G_0 is the base measure (i.e., an initial guess at the probability of success for each class) and G is a sample of Dirichlet distribution parameters (see equation 14a). We can think of DPs as "infinite-dimensional" Dirichlet distributions. We start from a finite mixture model and afterwards take the limit as the dimensionality approaches infinity. A mixture model is just another way of parameterizing the distribution and in the limit as the number of parameters approaches infinity, the model becomes nonparametric.

For a mixture of k components we have:

$$p(\tau|\boldsymbol{\pi}, \boldsymbol{\mu}) = \sum_{l=1}^{k} \pi_l K(\tau|\mu_l) \tag{11}$$

$$p(\mu) = G_0(\mu) \tag{12}$$

$$p(\pi_1, ..., \pi_k|\alpha) = \mathrm{Dir}\left(\frac{\alpha}{k}, ..., \frac{\alpha}{k}\right) = \frac{\Gamma(\alpha)}{\Gamma(\frac{\alpha}{k})^k} \prod_{l=1}^{k} \pi_l^{\frac{\alpha}{k}-1} \tag{13}$$

where the mixing proportions π_l must be positive and sum to one and Dir denotes the Dirichlet probability density function. Now we take the limit as $k \to \infty$. Samples G from a DP can be represented by a stick-breaking process [49], where a stick of length one is broken into k pieces, with π_1 denoting the length of piece one, π_2 the length of piece two and so forth:

$$G = \sum_{l=1}^{\infty} \pi_l \delta_{\mu_l} \tag{14a}$$

$$\mu_l \sim G_0 \tag{14b}$$

$$\pi_l = \beta_l \prod_{\lambda=1}^{l-1} (1 - \beta_\lambda) \ , \quad \beta_l \sim \mathrm{Beta}(\beta_l|1, \alpha) \tag{14c}$$

where $\mathrm{Beta}(x|a, b) = \frac{1}{B(a,b)} x^{a-1}(1 - x)^{b-1}$ is the beta distribution with normalization constant B. Thus the probability density for all mixture components $\boldsymbol{\pi}$ is:

$$p(\boldsymbol{\pi}|\alpha) = \int \prod_{l=1}^{\infty} \delta \left(\pi_l - \beta_l \prod_{\lambda=1}^{l-1} (1 - \beta_\lambda) \right) \mathrm{Beta}(\beta_l|1, \alpha) \mathrm{d}\beta_l. \tag{15}$$

1.3.5 Posterior probability of the classification vector

Assuming a flat prior for the classification vector $P(\mathbf{c})$, the posterior probability $P(\mathbf{c}|\mathbf{D})$ of the classification vector $\mathbf{c}$ given the already observed data is proportional to the likelihood of the

classification vector marginalized over the delay distribution, $P(\mathbf{c}|\mathbf{D}) \propto p(\mathbf{D}|\mathbf{c})$.

$$\begin{aligned} p(\mathbf{D}|\mathbf{c}) &= \prod_{j=1}^{n} p(D_j|\mathbf{c}) = \prod_{j=1}^{n} \int p(D_j|\mathbf{c},\boldsymbol{\pi},\boldsymbol{\mu})p(\boldsymbol{\pi}|\alpha)\mathrm{d}\boldsymbol{\pi}p(\boldsymbol{\mu})\mathrm{d}\boldsymbol{\mu} & (16)\\ &= \prod_{j=1}^{n} \int p\left(D_j|\mathbf{c},\left\{\pi_l = \beta_l \prod_{\lambda=1}^{l-1}(1-\beta_\lambda)\right\},\boldsymbol{\mu}\right) \times \\ &\qquad \prod_{k=1}^{\infty} \mathrm{Beta}(\beta_k|1,\alpha)\mathrm{d}\beta_k G_0(\mu_k)\mathrm{d}\mu_k & (17)\\ &= \prod_{j=1}^{n} \left(\sum_{s=1}^{\lfloor t_j \rfloor} \delta_{d_s c(x_s),R_j} \sum_{l=1}^{\infty}\right. \\ &\qquad \left.\int \beta_l \left(\prod_{\lambda=1}^{l-1}(1-\beta_\lambda)\right) K(t_j - s|\mu_l) \cdot \prod_{k=1}^{\infty} \underbrace{\mathrm{Beta}(\beta_k|1,\alpha)}_{\alpha(1-\beta_k)^{\alpha-1}} \mathrm{d}\beta_k G_0(\mu_k)\mathrm{d}\mu_k\right) & (18) \end{aligned}$$

where we used (9), (14) and (10c) in the last line. Now we collect the terms that depend on β_k and perform the integration over β_k. For all $k > l$ the integral yields $\int \mathrm{Beta}(\beta_k|1,\alpha)\mathrm{d}\beta_k = 1$. Note that there is an infinite number of these terms due to the infinite number of mixtures, but this does not pose a problem. For $k = l$ the integral yields $\int \mathrm{Beta}(\beta_l|1,\alpha)\beta_l \mathrm{d}\beta_l = 1/(\alpha+1)$. Finally for all $k < l$ the integral yields $\int \mathrm{Beta}(\beta_k|1,\alpha)(1-\beta_k)\mathrm{d}\beta_k = \alpha/(\alpha+1)$. There are $l-1$ terms with $k < l$, summarizing the integration over all β yields:

$$\int \beta_l \left(\prod_{\lambda=1}^{l-1}(1-\beta_\lambda)\right) \prod_{k=1}^{\infty} \underbrace{\mathrm{Beta}(\beta_k|1,\alpha)}_{\alpha(1-\beta_k)^{\alpha-1}} \mathrm{d}\beta_k = \frac{1}{\alpha+1}\left(\frac{\alpha}{\alpha+1}\right)^{l-1}. \tag{19}$$

Integrating over all μ yields:

$$\int K(t_j - s|\mu_l) \prod_{k=1}^{\infty} G_0(\mu_k)\mathrm{d}\mu_k = \int K(t_j - s|\mu) G_0(\mu)\mathrm{d}\mu \tag{20}$$

where we made use of the fact that for $k \neq l$ the integral over all factors containing k is $\int G_0(\mu_k)\mathrm{d}\mu_k = 1$. Performing the summation over all terms involving l gives a geometric series

$$\sum_{l=1}^{\infty} \frac{1}{\alpha+1}\left(\frac{\alpha}{\alpha+1}\right)^{l-1} = \frac{1}{\alpha+1}\sum_{l=0}^{\infty}\left(\frac{\alpha}{\alpha+1}\right)^{l} = 1. \tag{21}$$

Hence the concentration parameter α cancels. Putting things together again (18) simplifies to

$$p(\mathbf{D}|\mathbf{c}) = \prod_{j=1}^{n} \sum_{s=1}^{\lfloor t_j \rfloor} \delta_{d_s c(x_s),R_j} \int K(t_j - s|\mu) G_0(\mu)\mathrm{d}\mu. \tag{22}$$

In the considered task, the learner cannot distinguish between a continuous or a discrete delay distribution based on the observed data. We could therefore switch to using a Dirac-function as our kernel, $K(t_j - s|\mu) = \delta(t_j - s - \mu)$. This results in the following simple expression for the posterior:

$$P(\mathbf{c}|\mathbf{D}) \propto \prod_{j=1}^{n} \sum_{s=1}^{\lfloor t_j \rfloor} \delta_{d_s c(x_s),R_j} G_0(t_j - s). \tag{23}$$

1.4 Policy Gradient

There are two broad classes of reinforcement learning algorithms: value-based methods and policy gradient algorithms. Value based methods, such as temporal difference learning, assume the Markov-property and can fail on non-Markovian tasks as already shown in [1], whereas policy gradient algorithms do not fail. Therefore we compared human performance to that of the policy-gradient algorithm GPOMDP [10].

We parameterized the policy as $P(D = D_j|S_i) = \frac{\exp(\theta_{ij})}{\sum_k \exp(\theta_{ik})}$ analogous to Eq. (7) in the supplementary text, where parameters θ_{ij} describe the preference for decision D_j when observing stimulus S_i. To maintain the analogy with the neural model, the stimulus is the currently presented image in the intermixed reward task, or the currently presented and the previously presented images in the switch-state task.

Parameters θ_{ij} were initialized at zero and adapted based on the gradient of the average reward according to [10]. The continuous time formulation consists of an eligibility trace E_{ij} that is updated whenever a decision D is made in response to stimulus S according to $E_{ij} \leftarrow E_{ij} + \frac{\partial}{\partial \theta_{ij}} \ln P(D|S)$ and decays exponentially in between decision times, $\tau_R \dot{E_{ij}} = -E_{ij}$. Upon occurrence of reward R the parameters are updated according to $\theta_{ij} \leftarrow \theta_{ij} + \eta R E_{ij}$ with learning rate η as the only free parameter.

These updates are analogous to Eqs. (4) and (5). Indeed, the plasticity rule of the spiking neural population is based on the described policy gradient procedure, hence their similar performance might not be totally surprising, but nevertheless demonstrates that a biologically realistic implementation of GPOMDP with spiking neurons is possible without suffering loss in performance compared to the purely algorithmic implementation.

1.5 Akaike Information Criterion

The Akaike Information Criterion [11] adjusted for finite sample sizes (AICc) is computed in two steps as:

$$AIC = n \cdot ln\Big(\frac{RSS}{n}\Big) + 2k$$
$$AICc = AIC + \frac{2k(k+1)}{n-k-1}.$$

Here, n is the sample size (i.e, the number of subjects), k is the number of free parameters in the model, and RSS is the residual sum of squares ($RSS = \sum_i (x_i - \hat{x}_i)^2$, where x_i are the human data and $\hat{x}_i$ are the model predictions).
